# Delayed-onset parkinsonism is common after isolated striatal infarcts

**DOI:** 10.3389/fneur.2025.1653832

**Published:** 2025-11-20

**Authors:** Hedda Holm, Espen Dietrichs, Juho Joutsa, James P. Connelly, Christian G. Lund, Mona Skjelland, Vidar Gundersen, Silje Bjerknes

**Affiliations:** 1 Department of Neurology, Oslo University Hospital, Oslo, Norway; 2 Institute of Clinical Medicine, University of Oslo, Oslo, Norway; 3 Turku PET Centre, Neurocenter, Turku University Hospital, Turku, Finland; 4 Turku Brain and Mind Center, Clinical Neurosciences, University of Turku, Turku, Finland; 5 Department of Radiology and Nuclear Medicine, Oslo University Hospital, Oslo, Norway

**Keywords:** movement disorders, basal ganglia, corpus striatum, parkinsonian disorders, brain infarction

## Abstract

**Background:**

While case studies have suggested that only a minority of patients with putaminal lesions develop parkinsonism, existing data are limited by involvement of adjacent brain regions, brief follow-up period, and lack of systematic imaging. As a result, the true incidence and nature of parkinsonism following isolated striatal infarcts remain unknown. This study aimed to assess the incidence, timing, and clinical features of parkinsonism following isolated striatal infarcts using comprehensive imaging and longitudinal clinical assessments.

**Methods:**

We conducted a prospective cohort study at Oslo University Hospital, including patients treated with intravenous thrombolysis and/or mechanical thrombectomy for acute ischemic stroke resulting in isolated striatal infarcts. Patients with NIHSS scores of six or above were excluded to reduce the influence of other neurological deficits. Clinical evaluations included NIHSS, MDS-UPDRS, and MoCA scales at 3 months and 1 year. Brain MRI was performed at 3 months and [^123^I]FP-CIT SPECT imaging at 1 year to assess dopaminergic integrity.

**Results:**

Fifteen patients (median age 61) with unilateral striatal infarcts were included between June 2020 and January 2023. The median NIHSS score was one at both follow-ups. By 3 months, 27% (4/15) of patients developed parkinsonism, increasing to 67% (10/15) at 1 year. MDS-UPDRS motor scores showed a progressive increase over time, with contralateral akinetic-rigid symptoms predominating. Cognitive performance remained stable, with no significant changes in MoCA scores. Both the volume and location of the infarct appeared to influence the likelihood of developing motor symptoms. All patients showed reduced [^123^I]FP-CIT uptake in the infarcted striatum.

**Conclusion:**

This is the first study to systematically investigate delayed-onset parkinsonism in patients with isolated striatal infarcts, and our findings indicate that it may occur more frequently than previously recognized. These results challenge existing assumptions and highlight the potential value of repeated, targeted assessments in this population to improve detection and management of post-stroke parkinsonism.

## 1 Introduction

While it is widely known that lesions of the nigrostriatal tract can cause parkinsonism (1–3), there are a number of observations suggesting that putaminal lesions alone most often are not sufficient to cause parkinsonism, questioning the validity of the current models of the nigrostriatal tract in parkinsonism. In an influential meta-analysis among patients with putaminal lesions (all bilateral), only 2/20 patients had parkinsonism (4). In another study, which included nine patients with isolated putaminal infarcts, no patients exhibited parkinsonism when assessed during their hospital stay (5). Another study, however, found that in patients with CT findings of lacunar infarcts in either the caudate, lentiform nucleus or both, 17/45 patients (38%) had developed parkinsonism (6). In 2004, a study investigated the incidence of parkinsonism associated with striatal infarcts, focusing on lesions larger than 1.5 cm involving the basal ganglia and internal capsule (7). Here, the patients were examined on one occasion (5–15 months after the stroke), and only 1/11 patients developed parkinsonism.

Previous studies have faced several challenges. First, spontaneous striatal stroke lesions most often extend to the surrounding structures, and spontaneous isolated striatal strokes are rare. Lesions involving the structures surrounding the striatum might cause other symptoms, such as sensorimotor paresis and dystonia, which can complicate clinical evaluation or mask parkinsonism altogether. Second, post-stroke movement disorders have been reported with highly variable delays after the occurrence of stroke, and parkinsonism might not yet be present if assessing the patient too early. Third, most of the earlier studies were conducted decades ago when high-field strength MRI was not widely available, limiting the accuracy of stroke lesion localization. Finally, there are only a handful of cases of post-stroke parkinsonism scanned with molecular brain imaging techniques that allow for verification of a resulting striatal presynaptic dopamine defect (7, 8).

The modern era in neurology has opened new opportunities for studying isolated striatal infarcts. With the expanding use of intravenous thrombolysis (IVT) and mechanical thrombectomy (MT), more patients are likely to experience isolated striatal lesions. The basal ganglia are supplied by lenticulostriatal perforating arteries, which are terminal arteries branching from the proximal middle cerebral artery (9). The basal ganglia therefore lack collateral blood supply, making the striatum particularly vulnerable to ischemic damage. By the time reperfusion therapy is administered, an occlusion of the M1 segment of the middle cerebral artery can therefore cause irreversible striatal ischemia while sparing the cerebral cortex (10), resulting in isolated striatal lesions.

The aim of this study was to evaluate the incidence and clinical and imaging characteristics of parkinsonism following isolated striatal stroke lesions. We conducted a prospective, observational cohort study of patients who had an isolated striatal infarct following a middle cerebral artery occlusion with successful reperfusion therapy. The patients were followed up for a year after the occurrence of the stroke with repeated clinical assessments, brain MRI, and [^123^I]FP-CIT SPECT.

## 2 Methods

### 2.1 Study population

**Figure 1:**
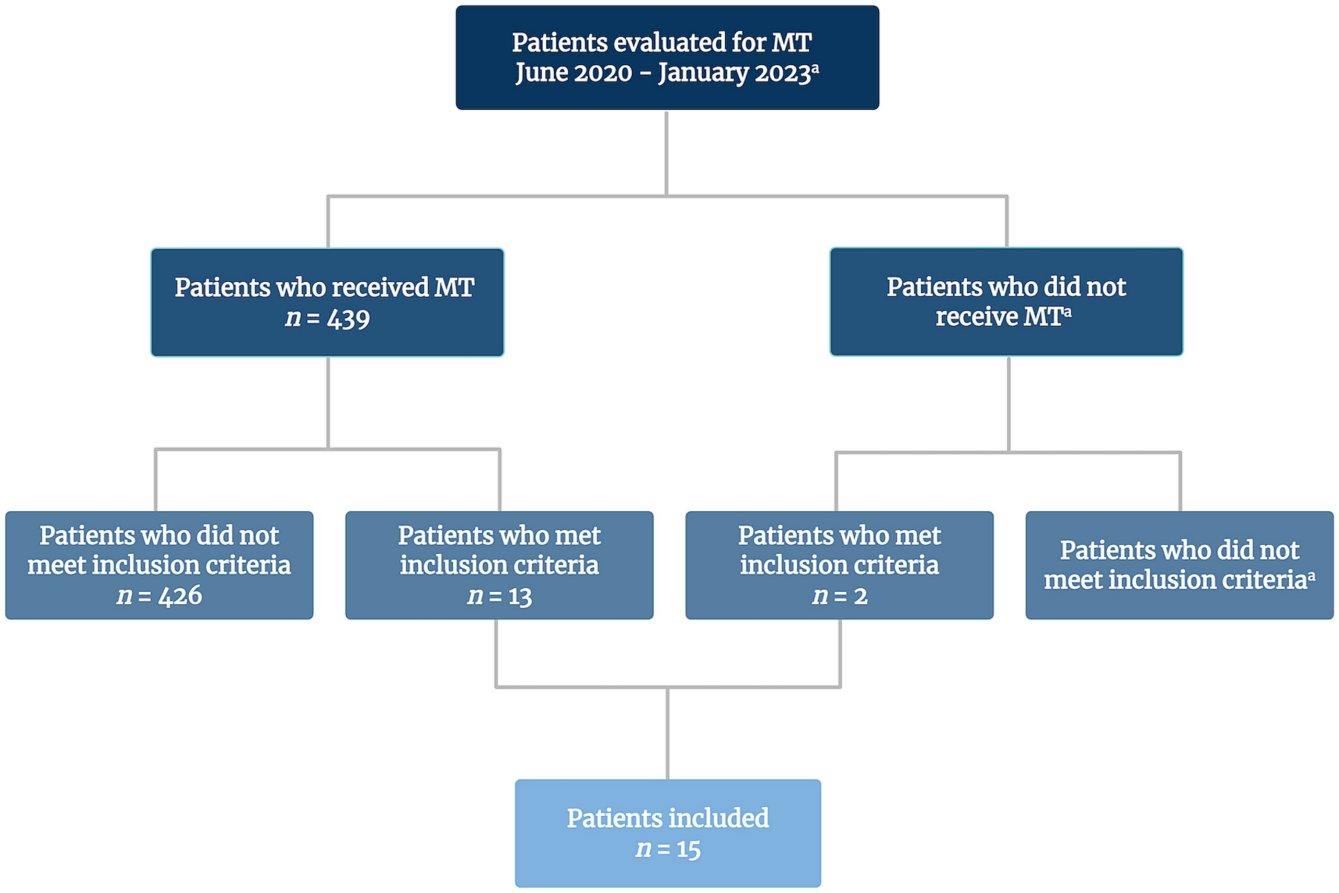
Patient flow diagram of study inclusion. MT, mechanical thrombectomy. ^a^Precise data on the total number of patients not available. Created in BioRender. Holm, H. (2025) https://BioRender.com/ixdskjs.

Study participants had suffered from acute ischemic stroke and were admitted for MT evaluation at Oslo University Hospital, Rikshospitalet in Norway, a regional thrombectomy center as well as a tertiary referral hospital for movement disorders. Patients enrolled in the study had a residual striatal lesion identified on T2-FLAIR weighted images from standardized MRI stroke protocol 1 day post-stroke. The enrollment period was from 1 June 2020 to 31 January 2023. During this period, 439 patients received MT, with or without prior IVT. Out of these, 13 patients met the inclusion criteria for the study. Two additional patients, who were evaluated for MT but ultimately received IVT alone or IVT in combination with intracerebral stent, also met the inclusion criteria. Thus, a total of 15 participants were included. A simplified patient flow diagram of the enrollment process is presented in Figure 1.

The exclusion criteria were: (i) infarcts extending into adjacent structures, e.g., the pallidum or internal capsule, cortical infarcts identified on T2-FLAIR weighted images, or extensive white matter lesions, (ii) score of six or above using the National Institutes of Health Stroke Scale (NIHSS) at discharge, as higher scores may reflect deficits that interfere with reliable motor examination, and (iii) any comorbidity that could confound the clinical or radiological evaluation.

### 2.2 Clinical evaluation

NIHSS (range 0–42) (11) was used to determine inclusion in the study. Neurological examination was then performed approximately 3 months and again 1 year following the acute stroke. The primary outcome of the study was diagnosis of parkinsonism, which required bradykinesia plus rest tremor and/or rigidity (12). While not a formal criterion, all patients with parkinsonism in this study exhibited bradykinesia with decrement, consistent with clinical parkinsonism. A movement disorder specialist verified the diagnosis.

Motor and non-motor symptoms of parkinsonism were further evaluated using the MDS Unified Parkinson’s Disease Rating Scale (MDS-UPDRS) (13). All examiners had completed the official MDS-UPDRS training and certification program. MDS-UPDRS part III (range 0–132) represents the motor examination and was conducted by two examiners, one of them a movement disorder specialist who was blinded to the clinical data and imaging results. Each examiner performed the assessment separately, after which a consensus score was reached. The MDS-UPDRS III score was also divided into bradykinetic-rigid symptoms (items 2–8 and 14), axial symptoms (items 1, 3 (neck), and 9–13), and tremor (items 15–18) (14). MDS-UPDRS part I and II (both range 0–52) concern non-motor and motor experiences of daily living, respectively. Cognition was assessed using the Montreal Cognitive Assessment (MoCA; range 0–30), which is more sensitive than Mini-Mental State Examination in detecting mild cognitive impairment (15), especially in vascular dementia (16) and in patients with parkinsonism (17). A commonly used MoCA cutoff score to indicate cognitive impairment is 26/30 (15). The test was conducted exclusively with patients whose native language was Norwegian.

### 2.3 MRI

1.5 T brain MRI was performed at three-month follow-up to measure lesion volumes. All measurements were conducted by the same examiner to ensure consistency in the analyses. T2-FLAIR weighted images were selected to measure lesion volumes, and images with slice thickness of 1 mm were uploaded to ITK-SNAP, a software application which provides semi-automatic segmentation and 3D volume calculation of structures in medical images (18). Following auto-segmentation, manual adjustment were made to refine the segmented region-of-interest. Infarct volumes were calculated based on the number of hyperintense voxels. We then calculated an infarct volume proportion that describes the proportion of the lesion volume relative to the total volume of the putamen, caudate, and striatum as a whole on the contralateral side. Due to varying image quality and natural variation in basal ganglia volume, the infarct volume proportion was used for statistical analyses rather than absolute infarct volume, so that each patient served as their own control. All imaging data were also reviewed and described by board-certified neuroradiologists as part of routine clinical assessment, providing an additional level of validation.

### 2.4 SPECT imaging

SPECT imaging was performed at 1 year using the same device for all study participants (Discovery 670 Pro). Nuclear medicine specialists conducted both the visual inspection of image quality and the semi-quantitative analysis. Specific [^123^I]FP-CIT binding was calculated by using region-to-occipital-cortex ratio for six striatal subregions (caudate, anterior and posterior putamen). The classification of patients to normal and abnormal [^123^I]FP-CIT binding groups was based on DaTQuant, a commercial software provided by GE HealthCare (United States), which enables automatic semi-quantitative calculations of striatal uptake. The software compares patient values to a normative database of 118 healthy controls (73 men, 45 women, age range 31–84 years) from the Parkinson Progression Markers Initiative. Abnormal ligand uptake was defined as equal or more than two standard deviations below the reference mean.

### 2.5 Statistical analyses

Stata 18 was used for statistical analyses. Analyses were performed using complete case methodology. That is, participants with missing data for a given variable were excluded from that specific analysis, and no data imputation was performed. A summary of the number of participants with available data for each clinical test and imaging modality at three-month and one-year follow-up is provided in Supplementary Table 1.

The primary focus of this study was descriptive. All hypothesis testing was conducted using the bootstrap t-technique with 10,000 replications, which resamples the original dataset to generate confidence intervals and *p*-values without relying on parametric assumptions (19). Only main, predefined comparisons were tested statistically; all other subgroup or exploratory analyses are presented descriptively with confidence intervals only, due to limited statistical power. Given the small number of prespecified tests, no correction for multiple comparisons was applied. Associations between continuous variables were examined using Pearson’s correlation coefficients, with bootstrap-derived *p*-values. All statistical tests were two-tailed, and *p*-values < 0.05 were considered statistically significant.

## 3 Results

Fifteen patients were included into the study and no patients were lost to follow-up (Table 1). All participants had been successfully treated for M1 occlusions, including five patients also treated for additional occlusions of the internal carotid artery and/or M2 segment. The median NIHSS score was 14 at admission (range 6–21) and two at discharge (range 0–5).

**Table 1:** Demographics.

Patient characteristics	Values
Age at baseline [yr in median (range)]	61 (43–74)
Sex [*n* (%)]	
Male	8/15 (53%)
Female	7/15 (47%)
Stroke treatment [*n* (%)]	
Only IVT	1/15 (6.7%)
Only MT	4/15 (26.7%)
IVT and MT	9/15 (60%)
IVT and intracerebral stent	1/15 (6.7%)
Affected cortical hemisphere [*n* (%)]	
Right	11/15 (73%)
Left	4/15 (27%)
Affected part of striatum [*n* (%)]	
Putamen	15/15 (100%)
Caudate	14/15 (93%)
Time of follow-up [d after stroke in median (range)]	
1st follow-up	103 (87–159)
2nd follow-up	383 (341–487)

d, days; IVT, intravenous thrombolysis; MT, mechanical thrombectomy; yr, years.

### 3.1 Parkinsonism and MDS-UPDRS III

**Figure 2:**
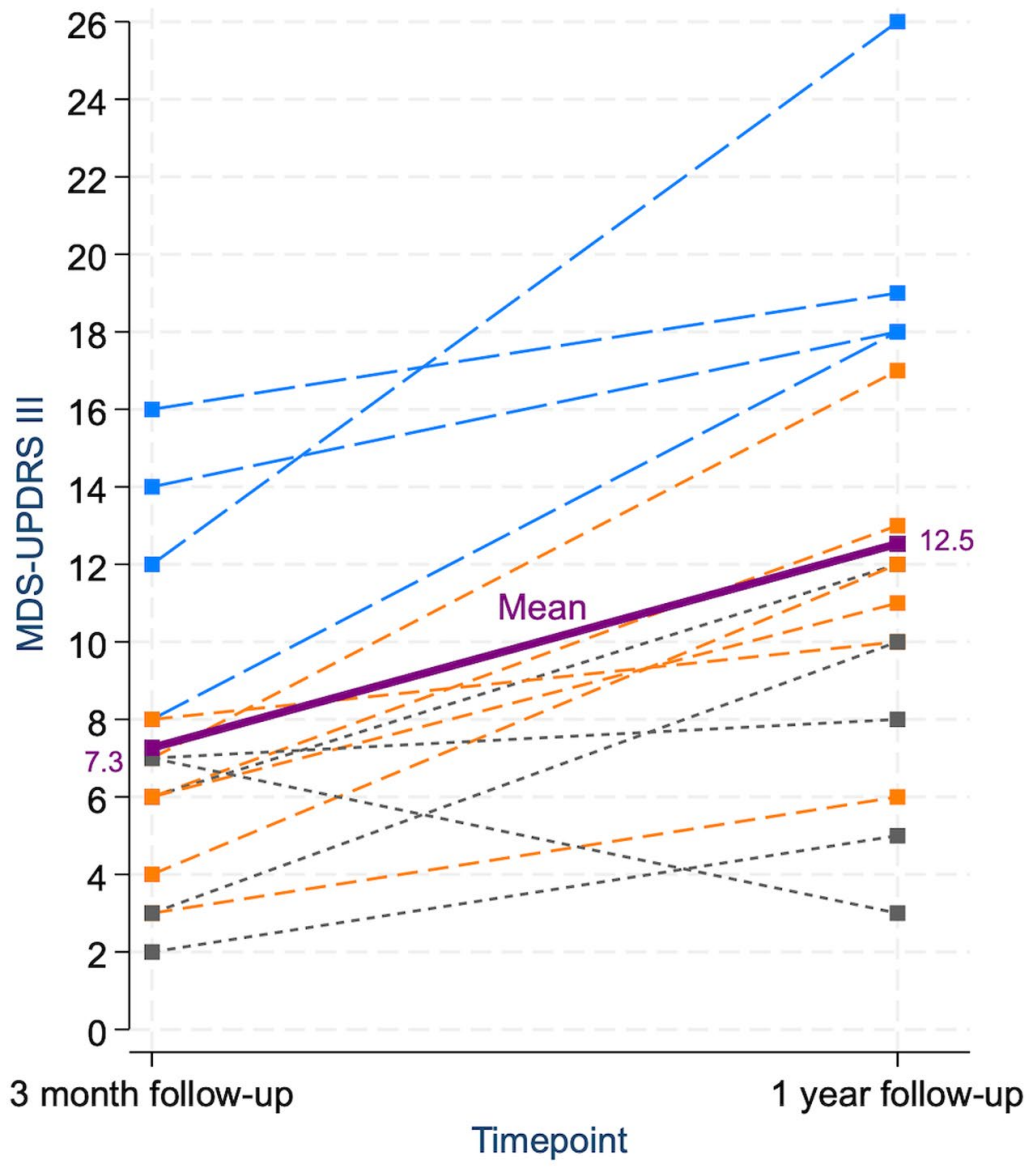
Individual and mean changes in MDS-UPDRS III scores from three-month to one-year follow-up. Each dashed line represents a single patient’s score trajectory. Patients with parkinsonism at both follow-ups are shown in blue, those who developed parkinsonism only at 1 year in orange, and those who never had parkinsonism in gray. The purple line and square markers represent the mean score at each timepoint. The increase in mean MDS-UPDRS III score from 3 months to 1 year was statistically significant. Only one patient showed a decrease in score; this patient exhibited isolated ipsilateral bradykinesia at 3 months, which had improved by 1 year, and never fulfilled criteria for parkinsonism.

At three-month follow-up, 4/15 patients (27%) had developed parkinsonism. By one-year follow-up, the prevalence of parkinsonism had increased to 10/15 (67%; Table 2). Patients who developed parkinsonism by 3 months were older than those who did not, but there was no age difference between the groups at 1 year. Across the whole cohort, the mean MDS-UPDRS III score increased significantly from three-month to one-year follow-up (*p* < 0.001; Figure 2). The score was notably higher in the parkinsonism group compared to the non-parkinsonism group at both time points. In the four patients with parkinsonism already at three-month follow-up, the MDS-UPDRS III score rose substantially from 3 months to 1 year. For the five patients who did not develop parkinsonism by the end of the follow-up, there also was a slight increase in the MDS-UPDRS III scores (Table 3).

**Table 2:** Distribution of parkinsonism.

Parkinsonism characteristics	3 mos [*n* (%)]	1 yr [*n* (%)]
Incidence of parkinsonism	4/15 (27%)	10/15 (67%)
Symptom type in patients with parkinsonism		
Rigidity	3/4 (75%)	10/10 (100%)
Rest tremor	1/4 (25%)	2/10 (20%)
Symptom side compared to the lesion in patients with parkinsonism		
Contralaterally	4/4 (100%)	9/10 (90%)
Bilaterally	0/4 (0%)	1/10 (10%)
Body parts involved in patients with parkinsonism		
Only upper extremities	3/4 (75%)	6/10 (60%)
Upper and lower extremities	1/4 (25%)	4/10 (40%)

mos, months; yr, year.

**Table 3:** Descriptive clinical characteristics in patients with and without parkinsonism at 3 mos and 1 yr.

Clinical characteristics	3 mos	1 yr
Age at baseline [median (range)]		
Parkinsonism	*n* = 4 73 (67–77)	*n* = 10 61.5 (47–77)
No parkinsonism	*n* = 11 57 (43–72)	*n* = 5 61 (43–72)
MDS-UPDRS III score [mean (95% CI)]		
Whole group	7.3 (5.0–9.5)	12.5 (9.1–15.9)
Parkinsonism vs. no parkinsonism		
Parkinsonism	12.5 (7.1–17.9)	15.0 (10.9–19.1)
No parkinsonism	5.4 (4.0–6.7)	7.6 (3.1–12.1)
Parkinsonism and no parkinsonism consistently		
Parkinsonism consistently (*n* = 4)	12.5 (7.1–17.9)	20.3 (14.1–26.4)
No parkinsonism consistently (*n* = 5)	5.0 (2.1–7.9)	7.6 (3.1–12.1)
MDS-UPDRS III symptom subscores [mean (95% CI)]		
Bradykinesia + rigidity		
Parkinsonism	10.0 (7.5–12.5)^a^	11.1 (9.0–13.2)
No parkinsonism	4.9 (3.5–6.3)	6.6 (3.5–9.7)
Axial symptoms		
Parkinsonism	2.7 (0–5)^a,b^	2.9 (0–6)^b^
No parkinsonism	0.5 (0–3)^b^	0.8 (0–3)^b^
Tremor		
Parkinsonism	1.3 (0–4)^a,b^	1.0 (0–5)^b^
No parkinsonism	0.0 (0–0)^b^	0.2 (0–1)^b^
MDS-UPDRS III score contra- and ipsilaterally to lesion in parkinsonism [mean (95% CI)]		
Contralaterally	9.3 (7–11)^a,b^	9.3 (7.5–11.1)
Ipsilaterally	0.7 (0–1)^a,b^	1.7 (0.6–2.8)
MDS-UPDRS III score in upper and lower extremities in parkinsonism [mean (95% CI)]		
Upper extremities	6.3 (2.5–10.1)^a^	7.1 (4.8–9.3)
Lower extremities	3.7 (0.8–6.5)^a^	4.0 (3.1–4.9)
MoCA score [mean (95% CI)]		
Whole group	28.0 (27.0–29.4)^a^	28.5 (27.4–29.5)^a^
Parkinsonism vs. no parkinsonism		
Parkinsonism	26.5 (23.7–29.3)	28.3 (26-30)^a,b^
No parkinsonism	29.1 (28.3–30.0)^a^	29.0 (28-30)^a,b^
NIHSS score [median (range)]		
Whole group	1 (0–2)	1 (0–2)

CI, confidence interval; mos, months; yr, year. ^a^Score not available for all patients; see Supplementary Table 1 for number of observations per variable. ^b^CI exceeded the instrument’s valid range. Values are therefore presented as mean (range) instead.

9/10 patients experienced hemiparkinsonism on the contralateral side, whereas one patient presented with bilateral parkinsonism at one-year follow-up, albeit with predominant symptoms contralaterally to the infarct. The MDS-UPDRS III score was significantly higher for the contralateral side in patients with parkinsonism at 1 year (*p* < 0.001; Table 3). Patients with parkinsonism primarily exhibited bradykinesia and rigidity, with tremor being rare (Table 2). Consequently, the MDS-UPDRS III scores were high for bradykinesia and rigidity but low for tremor and axial symptoms (Table 3). All patients with parkinsonism showed symptoms in their upper extremities, with some also showing additional symptoms in their lower extremities (Table 2). The MDS-UPDRS III score was significantly higher for upper compared to lower extremities (*p* = 0.001; Table 3). Among the four patients with parkinsonism at three-month follow-up, dopaminergic treatment was initiated in one patient. This anecdotal case did not experience improvement with levodopa/carbidopa 100/25 mg administered three times daily and opted not to try a higher dosage.

### 3.2 MDS-UPDRS I-II and cognition

For MDS-UPDRS I and II, there were no significant changes in scores between 3 months and 1 year (*p* = 0.278 and *p* = 0.559, respectively), and no marked differences between patients with and without parkinsonism at either time point (Table A1). At three-month follow-up, only one patient had a MoCA score one point below the cutoff score. However, by 1 year, all patients achieved scores above cutoff. The mean MoCA score for the entire group was 28/30 both at 3 months and 1 year, with no significant difference observed between these time points (*p* = 0.617). At 3 months, the four patients with parkinsonism had a lower MoCA score compared to patients without parkinsonism, but this difference was only minor at 1 year (Table 3).

### 3.3 MRI

All patients had unilateral infarcts in the posterior putamen on MRI, with partial involvement of the caudate in all except one patient (Table 1). The infarct volume proportion (proportion of infarct volume relative to the contralateral total nucleus volume) was significantly higher in the putamen compared to the caudate (*p* = 0.005). Patients with parkinsonism had higher infarct volume proportions in the putamen compared to those without parkinsonism, whereas no substantial difference was observed in the caudate. The infarct volume proportion in the putamen had a positive and stronger correlation with MDS-UPDRS III score at 1 year compared to the caudate, although neither correlation was significant. In addition, neither the infarct volume proportion of the putamen nor the caudate was significantly correlated with the MoCA score (Table 4).

**Table 4:** Lesion sizes and their association with clinical scores.

Groups	Infarct volume from total putamen volume [median (range)]	Infarct volume from total caudate volume [median (range)]
Whole group	45% (2–100)	19% (0–98)
Parkinsonism vs. no parkinsonism at 1 yr		
Parkinsonism	53% (5–100)	19% (9–71)
No parkinsonism	24% (2–97)	24% (0–98)

Clinical scores	Correlation with infarct volume from total putamen volume [*r* (95% CI)]	*p*-value (*r*)	Correlation with infarct volume from total caudate volume [*r* (95% CI)]	*p*-value (*r*)
MDS-UPDRS III score at 1 yr	0.39 (−0.29–0.80)	0.164	−0.02 (−0.51–0.45)	0.923
MoCA score at 1 yr^a^	−0.13 (−0.61–0.42)	0.618	−0.02 (−0.54–0.45)	0.930

CI, Confidence interval; *r*, correlation coefficient; yr, year. ^a^Score not available for all patients; see Supplementary Table 1 for number of observations per variable.

### 3.4 SPECT

**Figure 3:**
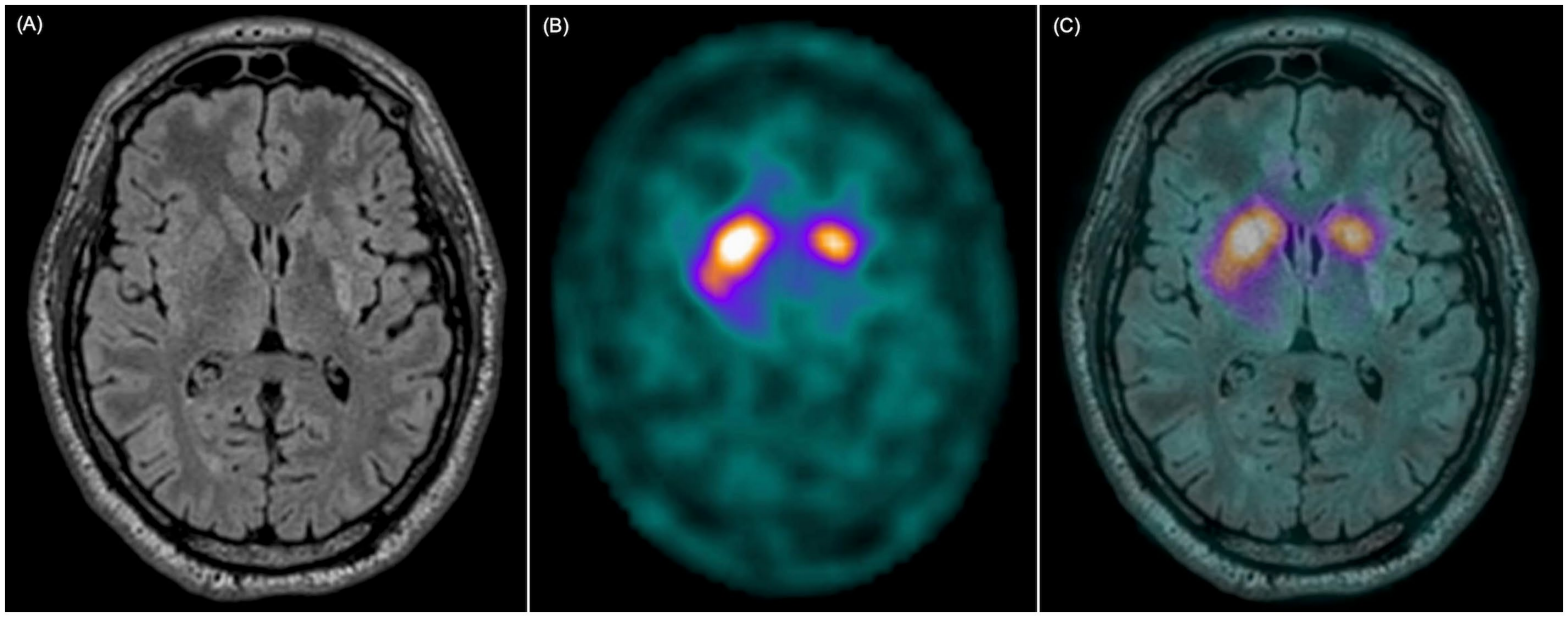
MRI and [^123^I]FP-CIT SPECT in a patient with an isolated striatal infarct. **(A)** T2-FLAIR weighted image shows infarct in the left posterior putamen. **(B)** [^**123**^I]FP-CIT SPECT shows reduced ligand uptake in the left posterior putamen. **(C)** Fusion of MRI and [^**123**^I]FP-CIT SPECT shows that the reduced ligand uptake closely follows the limits of the vascular lesion.

All patients showed reduced radiotracer uptake on [^123^I]FP-CIT SPECT at the stroke lesion site based on the visual evaluation (Figure 3). According to semi-quantitative regional analysis, 11/15 patients (73%) had abnormal SPECT imaging (Z-score ≤ −2 in at least one area), of which all included the posterior putamen. 8/10 patients with parkinsonism and 3/5 patients without parkinsonism at 1 year had abnormal semi-quantitative SPECT binding (Table 5). The Z-score was significantly lower on the infarcted side (*p* < 0.001), and it was lower in the putamen for patients with parkinsonism compared to those without (Table A2). There was a significant correlation between SPECT Z-score and infarct volume proportion for the striatum, indicating that the volume of the structural damage was coupled with the degree of dopamine deficit. However, there was no significant correlation between the MDS-UPDRS III score and striatal Z-scores for the striatum as a whole or for the posterior putamen (Table A3).

**Table 5:** [^123^I]FP-CIT binding.

[^123^I]FP-CIT binding	*n* (%)
Reduced binding based on visual evaluation	15/15 (100%)
Reduced binding based on semi-quantative analysis (*Z*-score)	
Posterior putamen	11/15 (73%)
Anterior putamen	4/15 (27%)
Caudate	4/15 (27%)
Reduced binding based on semi-quantative analysis (Z-score) for parkinsonism vs. no parkinsonism at 1 yr	
Parkinsonism	8/10 (80%)
No parkinsonism	3/5 (60%)

Yr, year.

## 4 Discussion

### 4.1 Interpretation of findings

There are several important findings in the present study. First, 27% (4/15) patients with isolated striatal infarcts developed parkinsonism by 3 months, increasing to 67% (10/15) by 1 year. Second, MDS-UPDRS motor score increased between 3 months and 1 year both in patients with and without parkinsonism at the first follow-up. Third, parkinsonism in these patients mainly manifested as contralateral akinetic-rigid symptoms in upper extremities, with only one patient with bilateral parkinsonism. Tremor and axial symptoms were rare. Fourth, the volume and location of the infarct appeared to influence the likelihood of developing motor symptoms. Although the putaminal lesion extended to the caudate in all but one of the 15 patients, none of the patients showed cognitive impairment, and there was no significant decline in MoCA scores in the one-year follow-up. Finally, all patients showed corresponding striatal dopamine deficit.

The incidence of parkinsonism following a striatal infarct was notably higher in our study compared to previous research. While 67% of our patients exhibited parkinsonism at 1 year, earlier studies reported incidences ranging from 0 to 38% (4–7). Several factors may explain this discrepancy. First, our study focused on patients with isolated striatal infarcts following successful IVT and/or MT, techniques that were not available at the time of the earlier studies. We used modern brain MRI rather than CT, which leads to more precise lesion localization (20). Notably, as our patients had isolated striatal infarcts and fulfilled inclusion criterion of NIHSS <6 (range 0–2), they did not exhibit motor impairments such as paresis or spasticity, minimizing the risk that parkinsonian symptoms were masked or confounded by other stroke-related deficits. In contrast, lesions in previous studies often extended to structures surrounding the striatum, potentially obscuring parkinsonism with other symptoms. Indeed, in one patient in the 2004 study, bradykinesia only became evident when the hemiparesis resolved (7). Second, the present study included repeated clinical examinations over a one-year period following the stroke, which may have enhanced the ability to detect parkinsonism compared to studies with shorter follow-up durations.

Our findings indicate that parkinsonism induced by isolated striatal lesions may follow a delayed and progressive course. Patients who did not meet the criteria for clinical parkinsonism at either of the time points still showed a slight increase in MDS-UPDRS III scores during follow-up (Figure 2). We hypothesize that these patients may develop clinical parkinsonism with a longer delay, but this remains to be confirmed by further follow-up.

In the chronic phase after an acute stroke, one might not typically expect further symptom development, in contrast to neurodegenerative diseases such as Parkinson’s disease (PD). Notably, the median NIHSS score was one (range 0–2) both at 3 months and 1 year, indicating that the symptom progression was not due to additional strokes. Possible explanations for the worsening of symptoms, however, is a retrograde denervation of nigrostriatal neurons and/or loss of compensatory mechanisms. Accordingly, a previous study showed degenerative changes in the substantia nigra 14 days after striatal infarcts (21), and the involvement of the nigrostriatal pathway is evidenced in the present study by the reduced striatal ligand uptake on [^123^I]FP-CIT SPECT. Similarly, numerous other studies have also demonstrated pre-synaptic dopaminergic dysfunction in patients with parkinsonism following infarcts involving the nigrostriatal tract (22–24). As we conducted SPECT imaging only cross-sectionally, repeated imaging in the future is needed to evaluate if the dopaminergic deficit is progressive and if this can explain the increasing symptoms in our patients.

Our patients primarily showed bradykinesia and rigidity, and to a much lesser extent tremor. This aligns with previous studies that have demonstrated that rest tremor is less frequent in patients with parkinsonism caused by cerebrovascular disease compared to PD. (25–28) Symptoms in our patients were mostly observed in the upper extremities, and gait disturbances were rare, apart from unilateral reduced arm swing in most patients. This pattern is consistent with what, by some, is termed vascular parkinsonism due to nigrostriatal lesions, as opposed to parkinsonism associated with extensive subcortical white matter lesions, which usually manifests with lower body symptoms and gait disorder (23). As expected, the majority of patients with parkinsonism developed symptoms contralaterally to the infarct. Only one patient, who did not exhibit signs of parkinsonism at 3 months, presented with bilateral symptoms at 1 year, including bradykinesia and rigidity in all four extremities, but still predominantly on the contralateral side. [^123^I]FP-CIT SPECT for this patient showed unilateral reduced ligand uptake corresponding to the vascular lesion. Notably, the one patient who developed parkinsonism in the 2004 study also exhibited bilateral symptoms with a unilateral infarct and corresponding unilateral reduced presynaptic dopamine function (7).

On MRI, the infarct volume in the putamen was higher in patients with parkinsonism compared to those without. Additionally, the proportion of infarct volume in the putamen moderately correlated with motor symptoms, while the caudate did not, although neither of these correlations reached significance. These findings align with the functional role of the striatal subregions: the putamen, particularly its posterior part, is well known to be activated during movement (29). In contrast, the caudate is more often associated with cognitive and affective function; however, based on the present findings, unilateral striatal infarcts affecting on average 1/4 of the caudate volume do not appear to be sufficient to cause clinically apparent cognitive deficits or progressive cognitive decline within 1 year after stroke.

All our patients had infarcts in the posterior putamen and reduced [^123^I]FP-CIT uptake in the same area (Figure 3), a region typically affected in PD. Although all patients showed reduced uptake on visual inspection, only 73% met the Z-score threshold for abnormality in DaTQuant. This discrepancy likely reflects the limitations of applying standardized Z-scores, developed for diffuse neurodegenerative conditions affecting entire anatomical regions, to focal infarcts involving only part of the striatum. As a result, DaTQuant may underestimate abnormalities in small, localized lesions such as those observed in this study. A representative case example illustrating this limitation is shown in Supplementary Figure 1. Notably, there was an apparent mismatch between imaging and clinical findings - i.e. reduced [^123^I]FP-CIT binding in some patients without clear parkinsonian symptoms. [^123^I]FP-CIT SPECT reflects presynaptic dopaminergic integrity, but not clinical expression, and a similar disconnect is well-documented in early PD, where abnormal scans can precede symptom onset (30, 31). In this context, the imaging confirms that the nigrostriatal pathway was structurally affected, even if clinical manifestations had not yet emerged. Longitudinal follow-up is needed to clarify whether these patients later develop symptoms.

The reason why some people developed parkinsonism by 1 year after stroke and others not, remains unknown. In this investigation, the development of parkinsonism may be related to infarct volume, particularly within the putamen. Additionally, patients who developed symptoms early were older, suggesting that increasing age might be a risk factor for lesion-induced parkinsonism. For PD, male sex and increased age are well-established risk factors, whereas alcohol, tobacco smoke, coffee drinking, and physical activity are factors that are associated with reduced risk of developing the disease (32). It is not established if these factors purely affect the process of neurodegeneration in PD, or if they also apply to parkinsonism caused by cerebrovascular disease. Therefore, we did not adjust for any factors in our study. Although all patients had infarcts in the posterior putamen and reduced ligand uptake on [^123^I]FP-CIT SPECT in the same area, another possible explanation for the different outcomes might be impairment of different brain networks based on slight differences in infarct localization. As the putamen is part of the nigrostriatal pathway and corticostriatal motor loops, it is possible that striatal lesion-induced parkinsonism is actually mediated via the functional connections disrupted by the lesion rather than the anatomical location of the infarct (33). Notably, one-third of patients did not develop parkinsonism, despite similar infarct localization and imaging findings. This may suggest the presence of individual or network-level resilience factors, which warrant further investigation.

### 4.2 Limitations

Our study is limited by its small sample size, and all analyses should be interpreted with caution. The findings require replication in larger cohorts to assess their generalizability. Still, the consistently high proportion of patients who developed parkinsonism provides proof-of-concept that isolated striatal infarcts can lead to delayed-onset parkinsonism. While comorbid PD cannot be entirely ruled out, it is unlikely to explain the findings in 10/15 patients. This is supported by the correspondence between symptom laterality and lesion location, the close match between reduced [^123^I]FP-CIT uptake and the infarcted area, and the absence of a typical PD pattern - namely, bilateral asymmetric binding deficits with a posterior-to-anterior gradient. Furthermore, the clinical assessment relied on subjective scoring systems; however, to reduce bias, two examiners independently performed the MDS-UPDRS part III and agreed on a final score. Cognitive function was assessed using the MoCA, which, although widely used, does not provide a comprehensive evaluation of cognition and may overlook subtle or domain-specific impairments such as executive dysfunction, which is often associated with frontostriatal circuits (29). Future studies may include more targeted neuropsychological testing to assess these deficits. Lastly, some patients with isolated striatal infarcts might have been missed during enrollment, and the reported number should not be interpreted as representative of their true frequency in this population.

Another limitation is the measurement of infarct volume on MRI. This has several potential sources of error, such as suboptimal image quality and indefinite criteria for ischemic changes, and these findings should therefore be interpreted with caution.

### 4.3 Clinical implications

We believe the expanding use of IVT and MT (34, 35) will lead to an increase of isolated striatal infarcts, as the area is particularly vulnerable to ischemic damage and may be irreversibly damaged by the time reperfusion therapy is administered (10). Furthermore, with the decline in stroke mortality (36), more patients are prone to experience post-stroke complications, including movement disorders (37).

Based on our results, we suggest that the current approach to patients with striatal infarcts could merit reconsideration. Historically, limited attention has been given to screening patients with isolated striatal infarcts for parkinsonism due to the belief that it rarely develops. Our data, however, suggest that these patients may be at high risk of developing delayed, progressive parkinsonism and could benefit from targeted assessments for parkinsonism during both the subacute and chronic phase. This might prevent misdiagnosis, enhance patient understanding of their symptoms and prognosis, and enable them to explore treatment options, such as levodopa. Moreover, they may benefit from tailored physiotherapy and multidisciplinary follow-up, akin to what is increasingly provided to patients with idiopathic PD and atypical parkinsonism syndromes (38). Importantly, the results are not intended to generalize to broader stroke populations, as lesions involving structures adjacent to the striatum may lead to other motor deficits, potentially masking or confounding the clinical assessment of parkinsonism.

Finally, this study contributes to the understanding of potential pathophysiological mechanisms underlying parkinsonism following cerebrovascular disease, providing insights into a complex and less well-characterized area. These observations may serve as a foundation for further exploration of alternative treatment strategies. Future research should include larger sample sizes and consider applying lesion network mapping to investigate how striatal infarcts disrupt connected brain networks, combined with extended follow-up to better assess the progression of parkinsonism after isolated striatal infarcts.

## Data Availability

The raw data supporting the conclusions of this article will be made available by the authors, without undue reservation.

## Appendix

**Table A1:** MDS-UPDRS I and II at 3 mos and 1 yr.

Clinical scores	3 mos [mean (95% CI)]	1 yr [mean (95% CI)]
MDS-UPDRS I score		
Whole group	9.1 (4.4–13.8)^a^	8.1 (3.5–12.7)^a^
Parkinsonism vs. no parkinsonism		
Parkinsonism	9.3 (5–15)^a,b^	7.8 (0–17)^a,b^
No parkinsonism	9.0 (0–26)^a,b^	8.2 (0–24)^b^
MDS-UPDRS II score		
Whole group	5.1 (1.6–8.5)^a^	5.3 (2.1–9.0)^a^
Parkinsonism vs. no parkinsonism		
Parkinsonism	6.3 (2–11)^a,b^	5.8 (0–11)^a,b^
No parkinsonism	4.7 (0–20)^a,b^	4.2 (0–18)^b^

CI, Confidence interval; mos, months; yr, year. ^a^Score not available for all patients; see Supplementary Table 1 for number of observations per variable. ^b^CI exceeded the instrument’s valid range. Values are therefore presented as mean (range) instead.

**Table A2:** [^123^I]FP-CIT binding in striatal subregions on ipsi- vs. contralateral side to the lesion.

Area	Z score [mean (95% CI)]	
	Ipsilateral to the lesion	Contralateral to the lesion
Striatum	−1.4 (−2.5 – −0.3)	1.4 (0.7–2.1)
Anterior putamen	−1.2 (−2.4 – −0.1)	1.2 (0.5–2.0)
Posterior putamen	−2.9 (−3.6 – −2.1)	1.0 (0.4–1.6)
Caudate	−0.5 (−1.8–0.8)	1.6 (0.8–2.5)
	Parkinsonism	No parkinsonism
Striatum	−1.5 (−3.1–0.2)	−1.3 (−3.2–0.7)
Anterior putamen	−1.4 (−3.1–0.3)	−0.9 (−3.1–1.3)
Posterior putamen	−3.1 (−4.1 − −2.2)	−2.3 (−3.7 − −0.9)
Caudate	−0.3 (−2.2–1.6)	−0.9 (−2.8–1.0)

CI, Confidence interval.

**Table A3:** Correlation with [^123^I]FP-CIT binding.

Imaging characteristics	Correlation with Z-score [*r* (95% CI)]	**Correlation with MDS-UPDRS III score at 1 yr [*r* (95% CI)]**	*p*-value (*r*)
Infarct volume proportion from total striatum volume	−0.73 (−0.88 – −0.44)		<0.001*
Z-score in different regions			
Striatum		−0.10 (−0.53–0.51)	0.703
Anterior putamen		−0.15 (−0.58–0.47)	
Posterior putamen		−0.19 (−0.60–0.31)	0.405
Caudate		−0.0048 (−0.44–0.64)	

CI, Confidence interval; *r*, correlation coefficient; yr, year. **p* < 0.001.
