# Supplementary figures and images for: Delayed-onset parkinsonism is common after isolated striatal infarcts
Source: Front Neurol. 2025 Nov 20;16:1653832. doi: 10.3389/fneur.2025.1653832 (PMC12676699; doi:10.3389/fneur.2025.1653832)

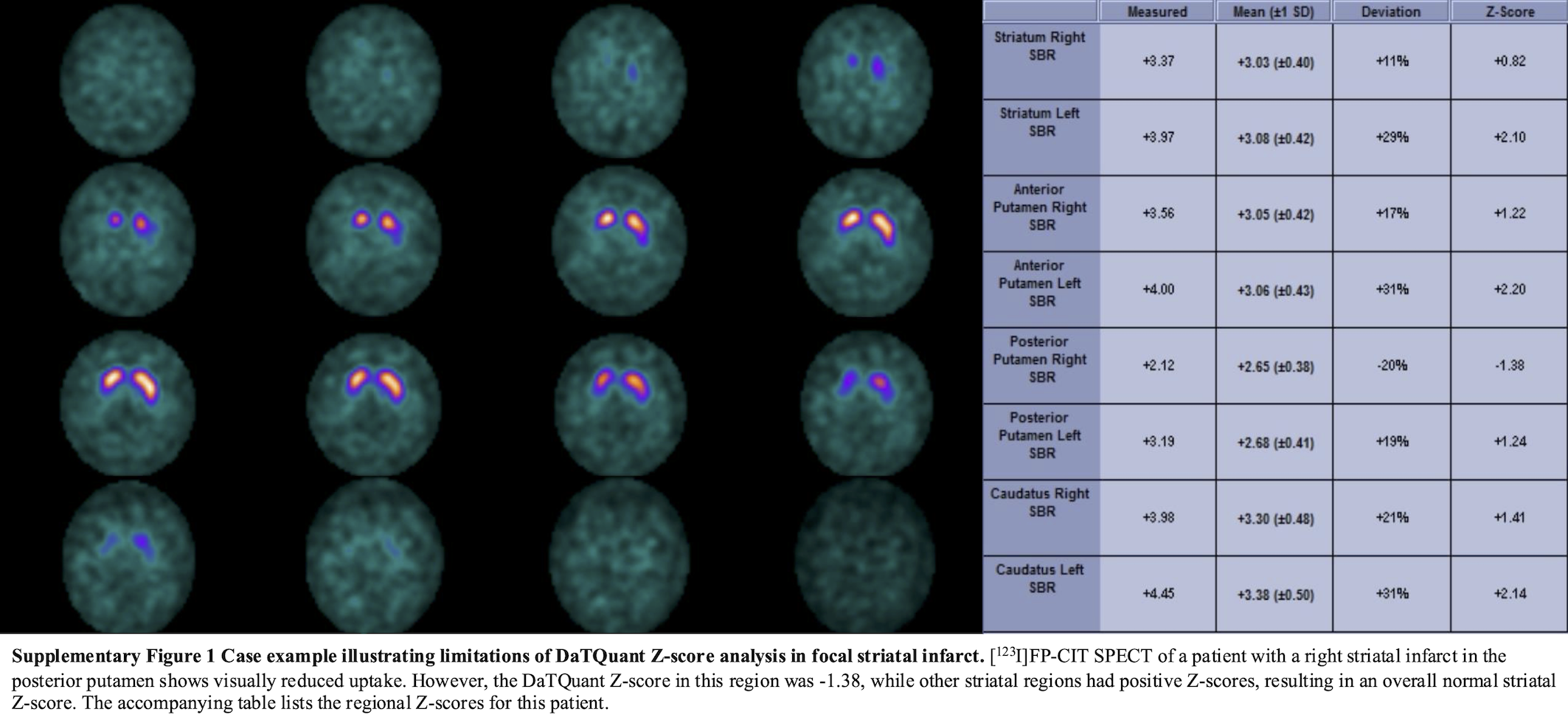

Supplement: Supplementary file 1 [file Image_1.TIFF]
