# Supplementary material for: Delayed-onset parkinsonism is common after isolated striatal infarcts
Source: Front Neurol. 2025 Nov 20;16:1653832. doi: 10.3389/fneur.2025.1653832 (PMC12676699; doi:10.3389/fneur.2025.1653832)
Supplement: Supplementary file 2 [file Table_1.docx]

**Supplementary Table 1 Number of participants with available data at 3 mos and 1 yr**

| **Clinical scores** | **3 mos [*n* (%)]** | **1 yr [*n* (%)]** |
| --- | --- | --- |
| **MDS-UPDRS III (full score)** | 15/15 (100%) | 15/15 (100%) |
| **MDS-UPDRS III (subscores)** | 14/15 (93%) | 15/15 (100%) |
| **MDS-UPDRS I** | 13/15 (87%) | 14/15 (93%) |
| **MDS-UPDRS II** | 13/15 (87%) | 14/15 (93%) |
| **MoCA** | 11/15 (73%) | 11/15 (73%) |
| **NIHSS** | 15/15 (100%) | 15/15 (100%) |
| **MRI** | 15/15 (100%) | N/A |
| **[^123^I]FP-CIT SPECT** | N/A | 15/15 (100%) |

mos, months; N/A, not applicable; yr, year
